# Grain Boundary Segregation in Pd-Cu-Ag Alloys for High Permeability Hydrogen Separation Membranes

**DOI:** 10.3390/membranes8030081

**Published:** 2018-09-12

**Authors:** Ole Martin Løvvik, Dongdong Zhao, Yanjun Li, Rune Bredesen, Thijs Peters

**Affiliations:** 1 SINTEF Industry, N-0314 Oslo, Norway; Rune.Bredesen@sintef.no (R.B.); Thijs.Peters@sintef.no (T.P.); 2 Department of Materials Science and Engineering, Norwegian University of Science and Technology (NTNU), 7491 Trondheim, Norway; dongdong.zhao@ntnu.no (D.Z.); yanjun.li@ntnu.no (Y.L.)

**Keywords:** membrane, hydrogen, palladium alloy, grain boundary

## Abstract

Dense metal membranes that are based on palladium (Pd) are promising for hydrogen separation and production due to their high selectivity and permeability. Optimization of alloy composition has normally focused on bulk properties, but there is growing evidence that grain boundaries (GBs) play a crucial role in the overall performance of membranes. The present study provides parameters and analyses of GBs in the ternary Pd-Ag-Cu system, based on first-principles electronic structure calculations. The segregation tendency of Cu, Ag, and vacancies towards 12 different coherent ∑ GBs in Pd was quantified using three different procedures for relaxation of supercell lattice constants, representing the outer bounds of infinitely elastic and stiff lattice around the GBs. This demonstrated a clear linear correlation between the excess volume and the GB energy when volume relaxation was allowed for. The point defects were attracted by most of the GBs that were investigated. Realistic atomic-scale models of binary Pd-Cu and ternary Pd-Cu-Ag alloys were created for the ∑5(210) boundary, in which the strong GB segregation tendency was affirmed. This is a starting point for more targeted engineering of alloys and grain structure in dense metal membranes and related systems.

## 1. Introduction

Cost-effective production of ultra-pure hydrogen can facilitate the widespread implementation of fuel cells and is one of the remaining bottlenecks before hydrogen can be introduced as an energy carrier on a large scale [1]. Dense metal membranes with high hydrogen permeance and selectivity have been identified as a promising enabling technology for efficiency improvement and cost reduction of hydrogen production. In particular, Pd-based hydrogen separation membranes are known to have 100% selectivity and high permeability, and thus allow for direct production of high purity hydrogen for use in fuel cells [2,3,4,5]. Combining these membranes with appropriate catalysts in membrane reactors to produce hydrogen from different sources has been described in numerous studies [1,6,7,8].

It appears that the potential of binary Pd-based membranes has been exhausted in the literature, and several groups have recently started working on ternary compounds as the next generation membrane material [9,10,11,12,13,14,15,16,17,18,19,20]. This has many possible benefits: the surface can be engineered to enhance the tolerance to impurity gases [13], the permeability can be optimized beyond what is possible with binary alloys [14], and the mechanical strength can be increased (e.g., if the self-diffusion is hindered or the morphology is changed) [2]. There is also potentially a cost reduction that is involved if expensive elements are replaced with cheaper ones [21]. The challenge with this approach is that ternary compounds are difficult to engineer when using plating, rolling, etc. as processing techniques [14]. One solution is to use a non-equilibrium process like magnetron sputtering to synthesize the active membrane material, which gives higher control of the material composition as well as the possibility of generating very thin membranes [14,22,23].

First-principles modelling has been demonstrated to be a powerful tool in the development of membrane materials [24]; as an example, density functional theory (DFT) has been used to systematically screen for novel binary intermetallic systems for hydrogen separation membranes [25]. A few studies have also investigated ternary alloys for membranes. The H_2_ permeability of the CuPd_1−*n*_*M_n_* system was studied by Sholl et al. [26,27] and Gao et al. [21], Pd-Ag-Cu by Ling et al. [17], while Løvvik et al. investigated sulfur adsorption on alloys in the Pd_1−*n*_Ag*M_n_* system [28].

Structural defects can play an important role in the kinetics of hydrogen in metals. It is well-known that hydrogen can segregate towards GBs in pure metals [29,30] and the local composition at GBs is thus of large interest for hydrogen permeation through dense metal membranes. Several DFT studies have investigated GB segregation in metals, e.g., in Ni [31], Ti [32], and Fe [33]. Other studies have focused on studying GB energies in alloys, e.g., in NiTi [34], Ti-Mo and Ti-V alloys [35], and in the ternary Ni_2_MnGa [36], and Ni-Al-Co systems [37]. Some studies have also investigated GB segregation in alloys, e.g., in Cu-Ag [38], various binary V alloys [39], and in the ternary Mg-Zn-Y [40] and FeCrNi [41] alloys. We are, however, not aware of any previous studies on GB segregation in Pd alloys that are based on first-principles calculations.

This work has investigated the effect of GBs on the distribution of elements in binary and ternary Pd alloys. This was done by studying increasingly complicated models, reflected by the structure of this paper. Initially, the properties of 12 different coherent ∑ GBs of pure fcc Pd will be presented. The emphasis is on properties that are relevant for segregation of defects, like the excess volume and bond range. A systematic investigation of the segregation tendency of three different defects in pure Pd will then be presented: single Ag and Cu solutes, as well as vacancies. This gives a general knowledge of defect segregation in the dilute limit and is potentially relevant for all alloys in the Pd-Ag-Cu alloy system. However, real Pd alloys display concentrations of e.g., Ag and Cu far beyond the dilute limit, so these results are not necessarily valid in realistic alloy systems. In order to corroborate this, the segregation tendency of Cu in a Pd-Cu alloy with 20–25% Cu is presented next. This Cu level was selected for the following reasons: Pd-Cu alloys have excellent sulfur tolerance [14], but exhibit rather low hydrogen permeability, except around the composition Pd_0.6_Cu_0.4_, where the crystal structure is bcc [12]. However, the bcc area at Pd_0.6_Cu_0.4_ is very narrow and difficult to obtain during preparation; the present study has therefore focused on the composition around Pd_0.8_Cu_0.2_, which has a good trade-off between sulfur tolerance and permeability. The last part investigates whether the segregation trends in the binary alloy systems hold when moving to ternary alloys. It is also focused on the region around Pd_0.8_Cu_0.2_, with Ag replacing Pd.

## 2. Methodology

**Figure 1:**
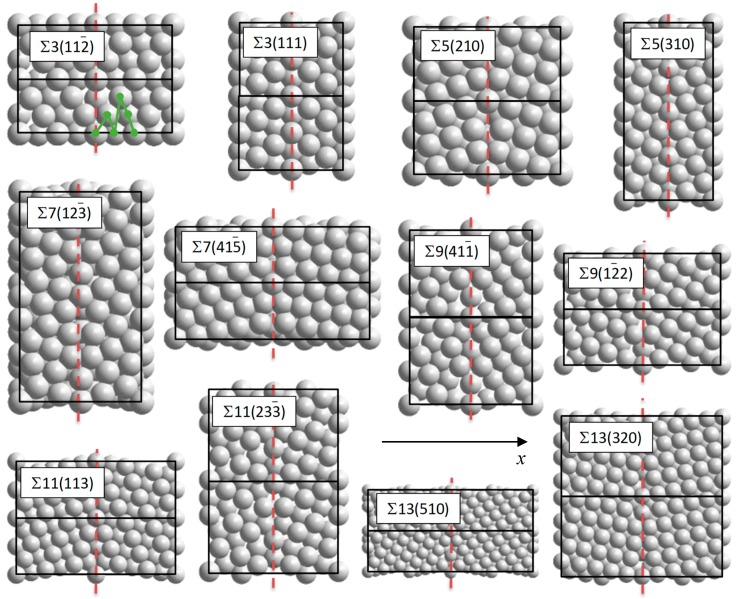
The different periodic grain boundary (GB) models included in this study. The dotted, red lines distinguish the GB planes. Unit cells are outlined with black lines, and the *x* direction is marked by the arrow. *x*_gb_ is defined as the horizontal distance from the GB planes. The supercell size perpendicular to the figure plane (the *z* direction) is listed in Table 1. The atomic positions plotted in Figure 3 have been shown as green dots connected by a solid line for the $\sum 3\left(11\bar{2}\right)$ model.

The calculations made use of the Vienna ab initio simulation package (VASP) [42,43], employing plane-wave basis functions with the projector augmented wave method [44] and the density functional theory (DFT) at the Perdew-Burke-Ernzerhof generalized gradient approximation (GGA) level [45]. The self-consistency requirement was changes in the total electronic energy less than 10^−5^ eV. The force relaxation criterion was 0.01 eV/Å, using the RMM-DIIS quasi-Newton method. Choosing a plane-wave cut-off energy of 500 eV and a k-point density of at least four points per Å^−1^ gave a numerical precision of better than 1 meV for relative total electronic energies. The GB models have been implemented in the supercell scheme, and the various supercells used are shown in Figure 1.

Three different schemes were employed as volume relaxation techniques (VRT): no relaxation, full relaxation of all cell parameters (including both cell size and shape), and only the relaxation of the cell length perpendicular to the GB plane (*x*). The atomic positions were relaxed in all the cases. Even if the present models do not physically resemble grains in a real material (the “grains” are sheets with infinite extension perpendicular to *x*), we can still learn about real materials by assessing how the different VRTs correspond to limiting cases of large or small domains in different directions. With this approach, the situation when only *x* is allowed to relax corresponds to the kind of relaxation that would find place for infinitely large grains, i.e., one single GB. With only one semi-infinite GB, the *x* direction will be fully relaxed, while the infinitely large lattice perpendicular to the GB is equivalent to an infinitely stiff lattice in the other directions. The relaxation along very large grains should thus be well represented by that found in our models when only *x* is allowed to relax. This option is not normally available in VASP but has been facilitated in a local version of the code. The relaxation procedures when either all or none lattice parameters are allowed to relax do not correspond directly to any physical distribution of GB in a real material. However, the two methods represent the outer limits of the kind of relaxation that would take place in a material with very small grains. In this case, relaxation perpendicular to any GB (corresponding to *x* in our models) will be countered by nearby GBs with other orientations. Similarly, relaxation along GBs will be partially allowed due to the finite size of the GB and the short distance to neighbor grains in all directions. Thus, the relaxation taking place in a material with very small grains should be in between the relaxation that is found when keeping the lattice fixed and when it is fully relaxed. We will in the following present results for all VRTs, hence describing relaxation effects that are likely to be seen in materials with large and small grain size.

## 3. Results

### 3.1. Structure and Stability of Pure Pd GB Models

Initially, the structure and stability of pure Pd GB models are investigated. The 12 different GBs investigated in this work are listed in Table 1. They are based on the coincidence site lattice model [46] and constitute all coherent tilt GBs with ∑ up to 13, thus exhibiting a quite wide range of deviations from the pure crystal. They have been represented by periodic atomistic models, as shown in Figure 1, where pure Pd models without point defects are displayed. These models range between 44 and 100 atoms (see Table 1), and their size in the *x* direction is between 13 Å (∑5(310)) and 40 Å (∑13(510)). Each model has two cancelling GBs: One at the unit cell boundary and one in the middle, identified by the dotted, red lines. The relatively large variation in size is partially due to symmetry (the models are often based on the smallest possible supercells with two such cancelling GBs) and the differing interaction range between various GBs—complex GBs typically display strain fields with larger range than simple ones.

Some inherent and calculated properties of the GB models are listed in Table 1. The excess volume per interface area $V_{X}$ is defined as the difference between the DFT relaxed unit cell volume and the corresponding volume of Pd atoms in the bulk, divided by the cross-section area *A* of the GB plane (perpendicular to *x*). The unit of *V_X_* is length and it can naively be interpreted as the accumulated extension of the lattice in the *x* direction due to the GB (when divided by 2, since there are two GBs per unit cell). The GB energy is defined as

(1) $$\gamma =\left(E\left(\mathrm{GB}\right)-N_{\mathrm{GB}}E\left(\mathrm{bulk}\right)\right)/2A$$

where the total energy *E* of the GB and of the bulk is that calculated by DFT, $N_{\mathrm{GB}}$ is the number of atoms in the GB model, and the factor 2 is due to the presence of two GBs in each unit cell. The bond range $∆r_{b}$ is the difference between the longest and shortest relaxed bond within the first coordination sphere of the atoms in each model. It is centered around the DFT equilibrium Pd-Pd distance of 2.80 Å with the shortest recorded relaxed bond distance being 2.43 Å and the longest one 3.46 Å; this gives $∆r_{b}$ up to 1.04 Å, which is seen for the ∑5(210) model. The distinction between the coordination spheres gets unclear for some of the models, and we have used a cut-off of 3.5 Å on the far side of this definition, which typically is a minimum between the first and second coordination spheres in these systems.

We first note that *V_X_* depends strongly on the VRT. This is not surprising, since the calculations with no volume relaxation are at the mercy of the initial model. When the unit cell is allowed to relax partially (only *x*) or fully, the spread in *V_X_* is smaller and the values of each model is consistent between the two procedures allowing for relaxation. *V_X_* is consistently smaller in the case of full relaxation, since a part of the volume expansion then is obtained within the GB plane. Keeping in mind that the only *x* relaxation corresponds to large grains while the relaxation of samples with small grains should be in between that of full relaxation and no relaxation, it appears that *V_X_* should be quite similar in magnitude, regardless of the typical grain size; the only *x* value is usually between the two others. The values vary between that of the ∑3(111) model (0.01–0.06 Å) and the $\sum 7\left(41\bar{5}\right)$ model (0.70–0.90 Å). These numbers may not be fully converged due to the limited size of the unit cells but are clear indicators of the deviation from perfect stacking. The former model can be viewed merely as a stacking fault, while the latter has quite large deviations from the perfect structure along the GB (see Figure 1).

**Figure 2:**
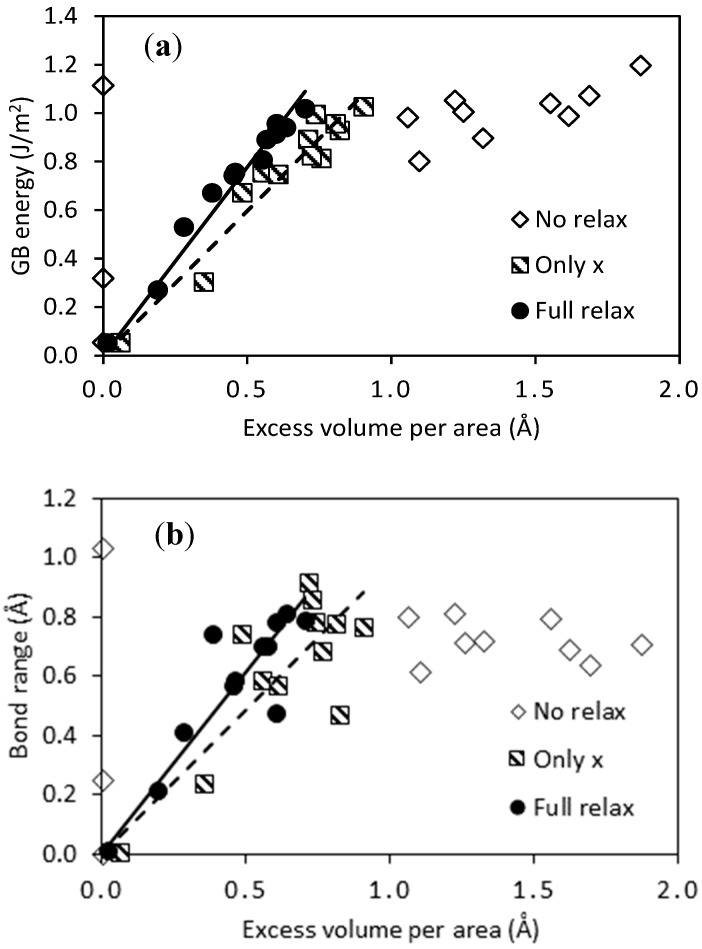
Relations between the excess volume per interface area *V_X_* and the GB energy *E*_GB_ (**a**) or the bond range $∆r_{b}$ (**b**) for the interface models listed in Table 1. The three VRTs are marked by open diamonds (no relaxation of the unit cell size), striped squares (only relaxation of the *x* axis), and filled circles (full relaxation of all degrees of freedom). Linear fits to the two latter techniques are shown as dashed and solid lines.

The same pattern is found when studying the GB energy γ; there is a clear correlation between *V_X_* and γ for the volume relaxed models, while the same is not the case when the unit cell was not relaxed. This correspondence is plotted in Figure 2a, showing that a very clear linear relation between *V_X_* and γ is obtained. The fit gives the empirical relation $\gamma =1.55\;V_{X}$ and $\gamma =1.19\;V_{X}$ for full and only *x* relaxation, correspondingly. The $R^{2}$ value is 0.97 and 0.93 for the two fits. This indicates that the volume misfit is an excellent predictor for the GB energy in the case of coherent defect-free GBs, which can potentially be used in experimental studies where *V_X_* may be easier to measure than γ. A similar correlation has been found previously for other materials, e.g., Ni [47].

The bond range $∆r_{b}$ is also following the excess volume and GB energy quite closely, if not as clearly as is the case between γ and $V_{X}$ (Figure 2b). Nevertheless, there is a clear correlation between $V_{X}$ and $∆r_{b}$—not surprising, since both parameters are a measure of the deviation from the perfect bulk crystal structure. It is perhaps more surprising that $∆r_{b}$ is lower when no volume relaxation is allowed than in the relaxed case, as is seen e.g., for the $\sum 3\left(11\bar{2}\right)$ model. This can be explained from the larger freedom of the volume relaxed models to accommodate strain by changing the local coordination within some of the models. Despite the spread in bond range and accompanying variation of coordination number (number of nearest neighbors) in the near vicinity of the GBs, all of the models display local atomic structures very close to fcc in between the GB regions. This ensures that close to bulk behavior can be found furthest away from the GBs in the models.

### 3.2. Segregation of Single Point Defects

**Figure 3:**
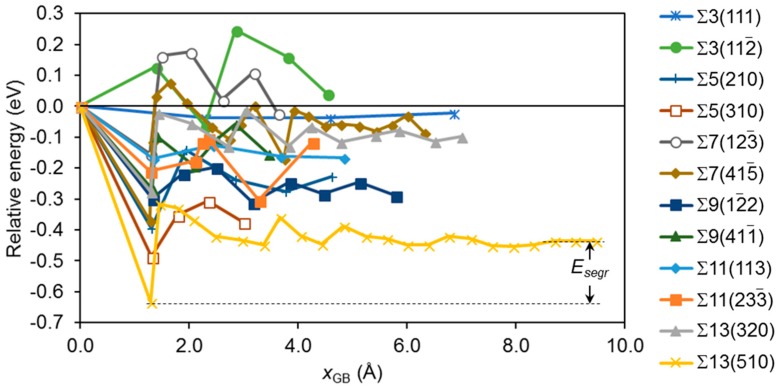
The relative energy *E*_gb_ in eV of a single Cu impurity Cu_Pd_ as a function of the distance *x*_gb_ to the plane of the ∑ boundary. The volume relaxation scheme was only relaxation of the *x* direction. Lines are drawn as guide to the eye. The segregation energy *E*_segr_ is taken as the difference between the average energy of the three sites furthest away from the GB and the lowest energy among the three nearest sites. The extension of a curve along the *x*_GB_ axis corresponds to half the size of the unit cell in the *x* direction. See the text for details.

The distribution of defects in the vicinity of GBs is determined by their relative energy at different positions and kinetics. We have only focused on energy in this work, since this is the most relevant in systems being allowed to equilibrate (which is the case in most membrane systems.) We have investigated three different defects with particular relevance for Pd membranes: substitutional Ag (Ag_Pd_) and Cu (Cu_Pd_), as well as vacancies (*V*_Pd_). This was done with the three different VRTs described above. The energy of each vacancy was calculated at various sites with increasing distance from the GB; an example of this is shown with the green curve for the $\sum 3\left(11\bar{2}\right)$ model in Figure 1. All the sites with unique *x* coordinates, from the GB to the midpoint between neighbor GBs were included (recall that cancelling GBs are present both at the unit size boundaries and at the red, dashed line.) The energy at the GB (*x* = 0) was used as the reference state, and the relative energy *E*_gb_ between that of the impurity located at *x* = *x*_gb_ and *x* = 0 was calculated for all 12 models. This is plotted in Figure 3 in the case of Cu_Pd_ defects with relaxation only along the *x* direction. Since the number of unique sites in the *x* direction differs between the various models, this is also the case with the number of plot points in Figure 3. The same applies to the distance between neighbor GBs, which is why the extension of the various curves varies in Figure 3. Similar plots to the one shown in Figure 3 have been generated for the three different VRT using three different defects (Cu impurities, Ag impurities, and vacancies); in total, nine plots. For simplicity the only relax *x* with Cu impurities is the only series of plots shown here.

In order to quantify the tendency to segregate towards or away from the GB the segregation energy of a specific defect has been defined as follows. The lowest total energy (as calculated by DFT) of the defect among the three sites nearest the GB is taken as “the” energy at the GB, *E*_GB_(def). This is compared to the average value of the three energies at the farthest distances away from the GB, defined as the “bulk” value *E*_bulk_(def). The segregation energy of the defect is then defined as

*E*_segr_(def) = *E*_GB_(def) − *E*_bulk_(def) (2)

An example is shown in Figure 3: the three values with highest *x*_gb_ (fitted with a black, dotted line) are used to calculate *E*_bulk_(Cu_Pd_) for ∑13(510), and the point at *x*_gb_ ≈ 1 Å (defining the lower black, dotted line) gives *E*_GB_(Cu_Pd_). The resulting segregation energy of this example (marked with black arrows in the figure) is *E*_segr_(Cu_Pd_) = −0.20 eV.

The model size should ideally be large enough for the energy to converge for large values of *x*_gb_. This can be seen for some of the models in Figure 3, but not all. Computational cost restricted the use of larger unit cells to achieve better convergence with respect to unit cell size. Nevertheless, some of the models exhibit excellent convergence as the *x*_gb_ increases. The ∑13(510) model is one example, where the energy does not change more than 0.03 eV when *x*_gb_ increases from 5 to 10 Å at 16 different sites, and the difference between the three sites with largest *x*_gb_ is less than 1 meV. Other models fluctuate more, but most of them exhibit a clear trend when *x*_gb_ increases. In some cases, there is no well-defined “bulk” energy; as an example, the energy of the ∑3(11$\bar{2}$) model varies significantly. This may be due to problems that are connected with the relatively small unit cells employed, and we have therefore in the following disregarded models where the average deviation from *E*_bulk_ is larger than 0.03 eV. Larger deviations than this are designating models with severe relaxation effects that are deemed as unphysical.

**Figure 4:**
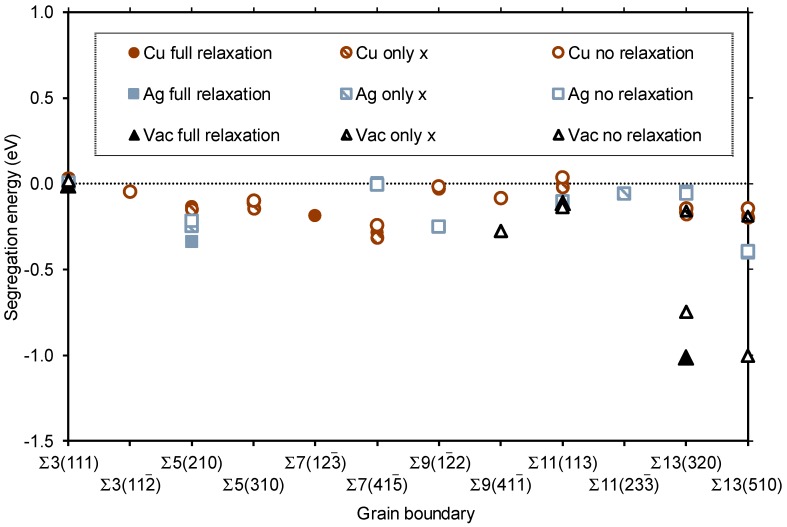
The segregation energy *E*_segr_ in eV of single Cu_Pd_, Ag_Pd_, and *V*_Pd_ defects for the various GBs. *E*_segr_ is shown for Cu (red circles), Ag (blue squares), and vacancy (black triangles) segregation, using full volume relaxation (filled symbols), relaxation along the *x* direction only (half-filled symbols), and no volume relaxation (open symbols). Only well-defined energies are included (see text).

The segregation energy was used to characterize the behavior of the three defects Cu_Pd_, *V*_Pd_, and Ag_Pd_ for all three VRTs and all 12 GB models; this has been compiled in Figure 4. A number of models displayed unphysical relaxations, and their *E*_segr_ values have not been included in the figure. This leaves part of the figure without data, but enough results were generated to draw some general conclusions. The most striking feature of Figure 4 is that almost all the segregation energies are negative, which indicates a tendency for all three defects to segregate towards the GB. The only small exceptions are for models where no volume relaxation was allowed, indicating that such relaxation is necessary in order to accommodate the defects at these specific GBs. The ∑3(111) twin boundary is special; since the GB is merely a stacking fault, there is nothing to gain from moving a point defect towards or away from the GB. *E*_segr_ is thus very close to zero for all defects and VRTs for ∑3(111). All other GBs exhibit significant values of *E*_segr_ for some or all defects and VRTs. The Cu_Pd_ defect gives the smallest absolute values of *E*_segr_ for most systems, indicating that the segregation tendency of Cu towards GBs in Pd is, in general, lower than that of Ag or vacancies.

**Figure 5:**
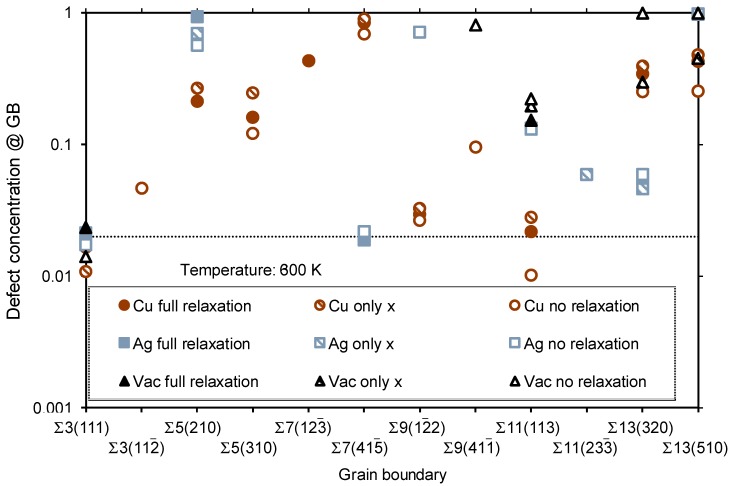
The defect concentration of point defects at the ∑ GBs listed in Table 1 and depicted in Figure 1 at *T* = 600 K. It is shown for the defects Cu_Pd_ (red circles), Ag_Pd_ (blue squares), and *V*_Pd_ (black triangles), using three VRTs: full volume relaxation (filled symbols), relaxation along the *x* direction only (half-filled symbols), and no volume relaxation (open symbols). The overall (bulk) defect concentration (chosen to be 0.02) is shown by the black, dotted line.

Based on the computed segregation energies the equilibrium concentration at the GB of a point defect *c*_def_ at a given temperature *T* can be found from the following equation [48]:

(3) $$c_{\mathrm{def}}=\frac{c_{\mathrm{def}}^{0}}{c_{\mathrm{def}}^{0}+\left(1-c_{\mathrm{def}}^{0}\right)\exp\left(E_{\mathrm{segr}}/k_{B}T\right)},$$

where $c_{\mathrm{def}}^{0}$ is the overall (“bulk”) equilibrium defect concentration, and *k_B_* is Boltzmann’s number. This formula assumes negligible interaction between solutes and thermodynamic equilibrium; we shall see later in this paper that the higher concentration of solutes may actually increase the anticipated equilibrium concentration in many cases. The resulting concentration has been shown in Figure 5 for the *V*_Pd_, Cu_Pd_, and Ag_Pd_ defects in Pd at *T* = 600 K, which is a relevant temperature for hydrogen separation membranes. The behavior is quite similar at higher temperatures (not shown), only with defect concentrations slightly closer to the bulk one (selected here to be 0.02). The negative segregation energies are reflected in defect concentrations significantly higher than $c_{\mathrm{def}}^{0}$ in most of the cases. In the example in Figure 3 (∑3(510)), this means that the site at layer 2 (directly next to the GB) has approximately 50% occupancy by the Cu solutes.

**Figure 6:**
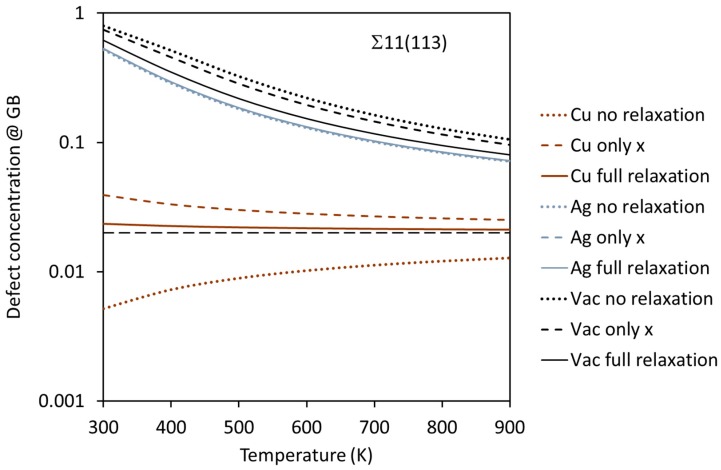
The defect concentration of point defects at the ∑11(113) GB as a function of temperature. The bulk equilibrium concentration (0.02) is shown as a black, long-dashed line.

The variation of the defect concentration with temperature has been depicted for the ∑11(113) GB in Figure 6. The concentrations approach the equilibrium bulk concentration of 0.02 (black, long-dashed line) as the temperature increases. The Cu impurity does not show a strong segregation tendency. In the case of only *x* relaxation (which corresponds to large grains) there is a weak segregation of Cu towards the GB. When no relaxation is allowed, however, there is a similarly weak segregation of Cu away from the GB. Since the situation with small grains corresponds to an interpolation between no relaxation and full relaxation, we conclude that Cu may be weakly segregated away from the GB in this case. Ag is, on the other hand, quite strongly segregated towards the GB and there is virtually no difference between large (only *x*) and small (between no relaxation and full relaxation) grains. Vacancies are also strongly attracted to the GB, and the difference between materials with large and small grain size should be quite small—interpolated values between the curves based on no relaxation and full relaxation are likely to be very similar to the values of the curve based on only *x* relaxed. We can deduce the temperature dependence of the other GBs in Figure 5 from the behavior of the curves in Figure 6, since the segregation energy is the only parameter that determines the defect concentration in Equation (3).

### 3.3. Binary Systems with More Than One Impurity Atom

**Figure 7:**
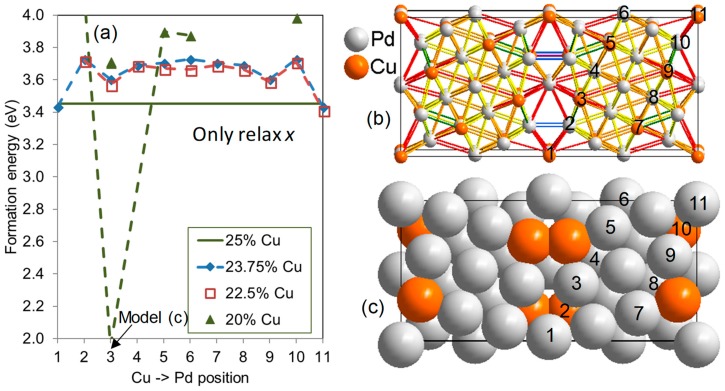
(**a**) The formation energy *E*_form_ of binary Pd_1−*n*_Cu*_n_* alloys with four levels of the Cu concentration *n* in a ∑5(210) GB model. The volume of all models is fully relaxed. The solid green line designating 25% Cu corresponds to the formation energy of perfectly ordered Pd_3_Cu as shown in (**b**). Lines between atoms are rainbow colored according to the relaxed interatomic distance; violet is shortest (<2.4 Å), dark red is longest (>2.9 Å), and orange is similar to that of density functional theory (DFT) relaxed bulk Pd_3_Cu (2.7 Å). Models with lower Cu content than 25% are generated by substituting Cu with Pd at various positions in the model, defined by the numbers in (**b**,**c**). The energy is drawn as a function of these positions. See the text for details about the formation energies plotted in (**a**).

Even if there is a clear tendency of segregation of impurities towards the GB in many GB models, it is unclear from the above whether more than one atom can be attracted to the GBs simultaneously. Repulsive interactions between point defects may lead to lower concentrations than that predicted in Figure 5. This was therefore investigated in more detail for Cu in Pd within a ∑5(210) GB model, as shown in Figure 7. The ∑5(210) GB was selected as a typical representative of the coherent GBs investigated in this study; it has a characteristic GB energy (~1 eV), range of bond lengths $∆r_{b}$ ~1 Å, as well as segregation energies of Ag (~−0.3 eV) and Cu (~−0.15 eV). This leads to a clear segregation behavior of solitary solutes with at least 21% (Cu) or 57% (Ag) equilibrium occupancy near the GB at 600 K when the overall concentration is 2%.

Since Cu and Ag segregate towards different sites, one can expect the situation of both solutes segregating simultaneously towards the same GB. To investigate this possibility a periodic GB model with stoichiometry Pd_3_Cu was generated as a starting point; a fully relaxed model is shown in Figure 7b. The bond lengths of the relaxed model are color coded in this figure. The relatively large variation of bond lengths with both elongated and contracted bonds explain why both large and small atoms may be attracted to the GB from a geometric point of view; this creates sites where atoms of various radii could be fitted geometrically. The bond lengths of this particular model vary from 2.31 to 3.29 Å, corresponding to $∆r_{b}$ = 0.98 Å. This is significantly larger than $∆r_{b}$ of the pure Pd ∑5(210) model, which only displayed bonds between 2.51 and 2.99 Å, thus $∆r_{b}$ = 0.48 Å (Table 1). This means that the addition of Cu not only decreases the smallest bond length (which can be expected when a smaller atom is added), but it also increases the largest one. This reflects a higher flexibility of the lattice when atoms with more than one size are present.

Models with the Cu content reduced below the starting point of 25% were created by replacing Cu by Pd in the model in Figure 7b. The stability of these models was assessed by their formation energy defined as

(4) $$E_{\mathrm{form}}\left({\mathrm{Pd}}_{N-n}{\mathrm{Cu}}_{n}\right)=E_{\mathrm{tot}}\left({\mathrm{Pd}}_{N-n}{\mathrm{Cu}}_{n}\right)-\left(N-n\right)E_{\mathrm{tot}}\left(\mathrm{Pd}\right)-nE_{\mathrm{tot}}\left(\mathrm{Cu}\right),$$

where $E_{\mathrm{tot}}$ is the total electronic energy as calculated by DFT, *N* is the number of atoms in the GB model (listed in Table 1), and *n* is the number of Pd atoms being substituted by Cu. The reference energy of Pd and Cu is that of their standard state, fcc bulk. Due to the difference in standard state energy between Pd and Cu, increasing the Pd content typically increases the formation energy (it appears less stable). The Cu content is reduced to 23.75% when one Cu atom is replaced, and the most stable configuration of this model is with extra Pd placed at position 1 or 11 (Figure 7a, dashed blue curve with diamonds), i.e., at the very center of the GB. The models with less Cu also exhibit the most stable configuration when excess Pd is placed at position 1 or 11. As an example, the most stable configuration of the 22.5% Cu model is with extra Pd placed at position 1 and 11 (dashed red curve with empty squares). The latter configuration is used as the starting point for the 20% model, which displays the most stable configuration with an extra Pd placed at position 3 or 9. A number of models with 20% Cu and Pd placed according to this insight were then constructed. The most stable of those is shown in Figure 7c, where all of the Cu atoms were moved to positions 2 and 10, corresponding to *E*_form_ = 1.9 eV. This is marked by the arrow at position 3 in (a). In conclusion, there is a very strong thermodynamic driving force for segregation of Cu towards the coherent ∑5(210) GB. From these results, it can be expected that all “small” sites (with short interatomic distances to the neighbor sites) close to this boundary are occupied by Cu. This can be quantified by $∆r_{b}$, which indirectly indicates the size of the smallest sites. From Table 1, it is evident that small sites exist in all of the models of this study (except the ∑3(111) twin boundary), and in many cases to a larger degree than in the ∑5(210) model. We can thus expect that the segregation trend of Cu is global, and that these results can be transferred to almost all GBs.

### 3.4. Segregation in Ternary Pd-Cu-Ag Alloys

The results of the above studies clearly show that there is a strong segregation of various point defects towards most GBs, and that this is valid even for a large density of solutes. However, it is not clear how different solutes interact with each other. From the size of defects as compared to the available sites near the GB (the “size effect”), one could expect that many GBs display a combined segregation of “small” and “large” defects (smaller and larger than the host atoms, respectively). We used a selection of Pd-Cu-Ag alloys with the composition ${\mathrm{Pd}}_{0.8-\delta }{\mathrm{Cu}}_{0.2}{\mathrm{Ag}}_{\delta }$ to investigate this hypothesis, again using the ∑5(210) GB as a representative model GB. The most stable models from Figure 7 were used as starting point, substituting Pd with Ag at various sites and concentrations.

The formation energy $E_{\mathrm{form}}$ of a Pd-Cu-Ag alloy is defined as similar to that of Pd-Cu in Equation (4):

(5) $$E_{\mathrm{form}}\left({\mathrm{Pd}}_{N-n-m}{\mathrm{Cu}}_{n}{\mathrm{Ag}}_{m}\right)=E_{\mathrm{tot}}\left({\mathrm{Pd}}_{N-n-m}{\mathrm{Cu}}_{n}{\mathrm{Ag}}_{m}\right)-\left(N-n-m\right)E_{\mathrm{tot}}\left(\mathrm{Pd}\right)-\left(n\right)E_{\mathrm{tot}}\left(\mathrm{Cu}\right)-\left(m\right)E_{\mathrm{tot}}\left(\mathrm{Ag}\right).$$

Here, *m* is the number of Pd atoms that have been substituted by Ag, and the reference energy of Ag is that of the standard state, fcc bulk. This definition means that a negative $E_{\mathrm{form}}$ indicates a stable compound compared to the pure metals. 

**Figure 8:**
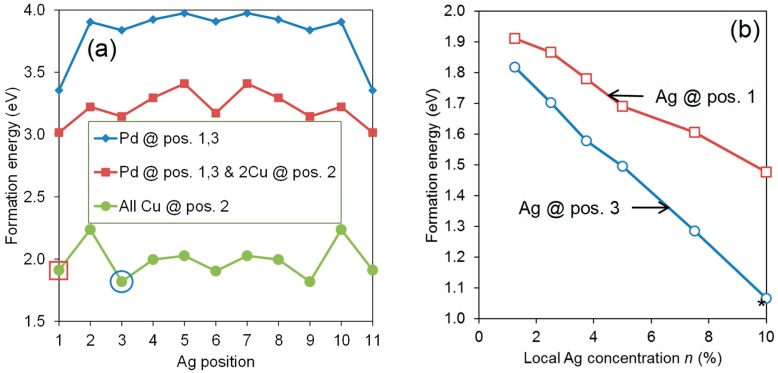
(**a**) The formation energy (defined in Equation (5)) of three fully relaxed ∑5(210) Pd_64_Cu_16_ models with Ag substituted at the sites defined in Figure 7c. One model was created from the periodic Pd_60_Cu_20_ model by substituting Pd for Cu at the positions 1, 3, 9, and 11 (blue line with diamonds), one had in addition moved two Cu atoms to positions 2 and 10 (red line with squares), and one had all Cu atoms located at positions 2 and 10 (green line with circles). The latter corresponds to the model shown in Figure 7c. The two most stable models from (**a**) were used to plot the formation energy as a function of local Ag concentration *n* in (**b**); Ag was then placed at position 1 (red circles) and position 3 (blue circles). The most stable model with *n* = 10% is shown in Figure 9b.

The formation energy is plotted for different models with Ag substituted for Pd or Cu as a function of the site (which corresponds to the distance from the GB) in Figure 8. We recognize the trend from the binary alloy; Ag is most stable at the GB (represented by the positions 1 and 11) in the model where Pd has replaced Cu at positions 1 and 3. It is also the most stable in the model where two Cu atoms in addition have been segregated to site 2 and 10, but to a smaller extent. However, in the most stable situation when all the Cu atoms are moved towards the GB and only populate sites 2 and 10 (corresponding to Figure 7c), the Ag site with lowest energy is not anymore at the GB (position 1 or 11), but rather at sites 3 and 9, which are just outside the Cu sites. This is due to relatively large local relaxations around the Cu atoms, which reduce the size of the sites at the GB (position 1 and 11). These sites are thus not significantly smaller than other sites anymore. Adding to this is the reduced affinity between Ag and Cu when compared to that between Ag and Pd. Since the latter model is the most stable one, we can expect to find the enrichment of Cu in position 2 and of Ag in position 3 close to ∑5(210) GBs in Pd-Ag-Cu compounds. This is supported by the plot in Figure 8b, demonstrating that the formation energy decreases as the local Ag concentration increases.

**Figure 9:**
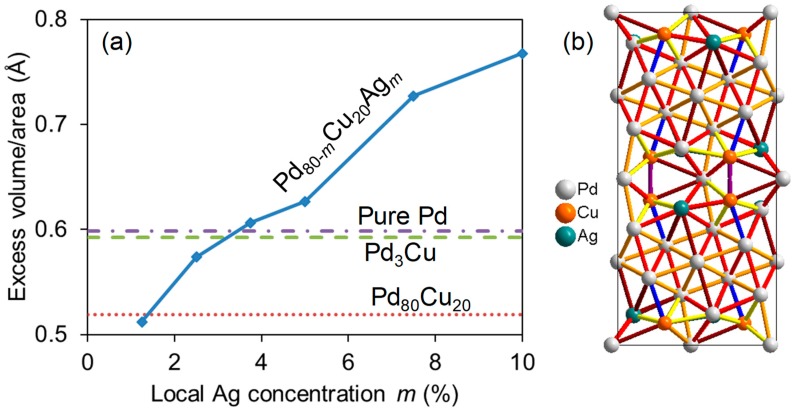
(**a**) The excess volume/area of an 80-atom ∑5(210) model (Figure 1) for pure Pd (dashed-dotted line), Pd_3_Cu (dashed), Pd_80_Cu_20_ (dotted), and Pd_80−*m*_Cu_20_Ag*_m_* (solid). The local Ag concentration *x* corresponds to the concentration very close to the GB and does not necessarily reflect the overall concentration (it may have segregated towards the GB). The most stable model with *m* = 10% is shown in (**b**), and the interatomic distances *d* are indicated with rainbow colors, starting from dark red (*d* > 2.9 Å) to violet (*d* < 2.5 Å). The average bond distance in bulk Pd is approximately 2.7 Å, represented by yellow (2.6 < *d* < 2.7 Å) and orange (2.7 < *d* < 2.8 Å) bonds.

How does the addition and segregation of Ag influence the bond distances at and near the ∑5(210) GB? This may be relevant for hydrogen solubility and diffusivity since both depend strongly on the interatomic distances. Figure 9 presents how this is quantified by the excess volume divided by GB area in Figure 9a and by actual bond distances in the most stable model with the composition Pd_70_Cu_20_Ag_10_ in Figure 9b. It is evident that an increasing amount of Ag at the GB leads to significantly increased excess volumes. Since the volumes are divided by the GB area (that of the relaxed unit cell), this primarily signifies the elongation of the GB unit cell in the *x* direction. The Pd-Pd-bonds in the near-GB region are thus clearly larger than those in the bulk Pd, and significantly more so than in the bulk Pd-Cu alloys, where the overall lattice constants are reduced due to the smaller size of Cu atoms.

## 4. Discussion

The 12 GB models depicted in Figure 1 are by no means representing all the possible GBs in fcc Pd, even when being restricted to coherent ones without compensating dislocations or other defects. The relevance of the present results is thus restricted to a selection of such interfaces, which may not govern all important properties of these compounds. Nevertheless, we have seen that the selection of models gives a broad distribution of important parameters, like the interface energy, mismatch volume, range of bond lengths, etc. This indicates that the present results should be relevant at least for all coherent GBs in Pd alloys, even those with lower angles than the present ones.

The GBs in this study are all perfectly coherent, which of course is a simplification of the real situation. Many of the GBs found in real materials are quite complex, featuring all sorts of defects that to some extent compensate geometric mismatches that are inherent to the perfect GB. Nevertheless, many of these additional defects can be relatively far apart (e.g., in the case of dislocations), leaving near-perfect GBs over large areas, as demonstrated by several microscopy studies [49,50]. We therefore assume that our coherent models may be also relevant for a number of GBs where the lack of coherence is not too severe. We expect the correspondence to fail when going to truly amorphous GBs.

The results above suggest significant segregation of a variety of point defects towards virtually all GBs. However, the absolute numbers in Figure 5, summarizing the segregation tendency, should be applied with some care due to a number of reasons. The limited size of the models means that the strain originating from the GBs is not converged to zero at any place in the super cell. This challenge has been accommodated to some extent by using the three different volume relaxation methods—they represent the outer limits of how the unit cells should realistically be relaxed in the vicinity of GBs, and the real segregation tendencies should be somewhere between those limits. So even if there is no true bulk behavior between the GBs in the various models, the local relaxation effects and the resulting segregation should be correct within the boundaries that are described by the different VRTs.

Another potential source of error in these calculations is the lack of structurally compensating defects, most notably dislocations. They can accommodate significant parts of local strain and could as such counter some of the strongest segregation tendencies that were seen in this study. However, dislocations can attract point defects themselves, so this does not necessarily hinder segregation of defects, even if the nature of the segregation might be changed.

Another reason to take the absolute numbers of Figure 4 with some care is the rather large relaxation effects that were seen for some of the models. In some cases, the entire supercell was restructured, which led to a total energy being reduced by several eV in some of the cases. This can be understood as the starting point of a full relaxation to the lowest energy structure, which is the bulk without any GB. We have disregarded the points with largest restructuring effects, but it was difficult to distinguish between reasonable relaxations and unphysical effects due to the small size of the supercells; there was a continuous range from virtually no relaxation to almost complete reorganization of the GB model. We disregarded models where the average deviation from *E*_bulk_ is larger than 0.03 eV, but some unphysical results due to limited unit cell size may still remain.

The calculations of the present study have all been performed without any explicit temperature being included. Temperature was included implicitly through the Arrhenius equation when obtaining equilibrium defect concentrations in Figure 5, but no other effect of temperature (thermal expansion, entropy, zero-point energy, phonon-based thermodynamics, etc.) was included. This has the potential to change the quantitative results significantly, but we expect that the qualitative trends remain unchanged.

Many of the same concerns apply when turning to the ternary compositions. We established that both larger (Ag) and smaller (Cu) atoms are attracted to a GB simultaneously, but the absolute value of the numbers and the actual sites of attraction may be different in reality than what is reported in this study. Furthermore, we did not consider simultaneous segregation of vacancies and solutes. With the knowledge from above, we expect that this is present in most boundaries, since vacancies and solutes have the ability to occupy different sites around a given GB.

The various sources of potential errors thus add up to a large and unknown uncertainty of the numbers presented in this study. The remaining main conclusion is unchanged, however: there is a clear and consistent tendency of many kinds of point defects (small and large substitutes, vacancies) to segregate towards GBs, due to the variety of local environments that are found there.

Despite the clear trends, it may be challenging to observe the segregation experimentally. It happens on the scale of single atomic layers, which makes high-resolution transmission electron microscopy the only viable way of directly probing such segregation. This relies on the ability to focus the electron beam along the GB plane, since this is where the change in concentration should be observed. It may also rely on the synthesis and heat treatment of the film; e.g., since sputtering is a non-equilibrium synthesis process, annealing (or operation under realistic conditions) may be required for the segregation to appear. Furthermore, Pd-membranes are typically aimed for hydrogen separation purposes, and the presence of hydrogen may influence the segregation behavior significantly, as has been seen in the case of segregation towards outer surfaces of similar systems [51].

Three different VRTs were compared for many of the calculations above, representing the boundaries of the likely volume relaxation regimes in real materials (from relaxation along *x* signifying low density of GBs to no relaxation representing very high density of GBs). However, some of the results indicate that not performing any relaxation of the volume is unreasonable. This is particularly clearly illustrated in Figure 2, where the linear trend between *V_X_* and γ can only be seen if the partial or full relaxation of the model is allowed. The conclusion from this observation is that some relaxation of the volume is required to move away from unreasonable situations arising from the somewhat arbitrary construction of the GB models. Most of the results in this study that are based on no volume relaxation should thus be neglected, apart from serving as a far-end borderline of the values.

Which of the VRTs allowing for volume relaxation to choose is less obvious. Both partial (only *x*) and full relaxation of the unit cell give results in reasonable correspondence with each other, indicating that relaxation of the coordinate perpendicular to the GB plane is most important. There are some differences between the two, depending on the particulars of the GB; most notably, the $\sum 11\left(23\bar{3}\right)$ GB exhibits large differences between only *x* and full relaxation, both for *V_X_*, γ, and $∆r_{b}$ (Table 1). This reflects that this particular GB displays significant relaxation of the unit cell parameters parallel to the GB plane when allowed, in contrast to the other GB models. Such relaxation is most reasonable when the real GB resembles our simulation cell: infinitely long in the directions parallel to the GB plane but with a short distance between the GBs in the *x* direction. Partial relaxation (only *x*) describes situations where the average bulk lattice constant is maintained in these parallel directions by an infinitely stiff lattice, i.e., extending far away from the GB.

## 5. Conclusions

Atomic-scale calculations based on density functional theory were used to investigate various properties of low-number coherent ∑ grain boundaries (GBs) in fcc Pd, Pd-Cu, and Pd-Cu-Ag alloys. Their excess volumes, grain boundary energies, and ranges of bond lengths were computed while using three different volume relaxation techniques: no relaxation of volume, full relaxation of all lattice parameters, and partial relaxation of volume only allowing for one lattice constant to change. A linear correlation between the excess volume and grain boundary energy was found in pure Pd when partial or full relaxation of the volume was allowed. The tendency to segregate towards the GBs was assessed for three different point defects: Cu_Pd_, Ag_Pd_, and *V*_Pd_. Virtually all GBs exhibited strong segregation tendencies for all defects, quantified by the segregation energy and the corresponding equilibrium concentration of the defects at or near the GBs. An amplified segregation tendency was observed when increasing the local solute concentration, indicating that many of these sites might be nearly fully occupied by substitutional defects. This also demonstrated that the initial study at the dilute limit is clearly relevant for alloys with higher concentrations of the alloying element. Ternary Pd-Cu-Ag compounds were finally investigated, and simultaneous segregation of both Cu and Ag towards the ∑5(210) GB was observed; i.e., the results also hold for more complex alloy systems. The most stable model furthermore displayed a pronounced increase in the excess volume, indicating significantly increased local lattice parameters. In summary, this study demonstrates how insight from first principles calculations can be used to understand the complex behavior of point defects at and around grain boundaries of metals and alloys.

## Figures and Tables

**Table 1:** The various grain boundary (GB) models investigated in this study, along with the number of atoms in their unit cells, the supercell size in the *z* direction $l_{z}$ (perpendicular to the figure plane in Figure 1), their excess volume divided by area *V_X_*, the calculated GB energy *E*_GB_, and the range of bond distances $∆r_{b}$ within the first coordination shell. All results are for pure Pd GBs. Three volume relaxation techniques (VRT) were used: no relaxation of the unit cell, only relaxation of the *x* lattice constant (see Figure 1 for a definition of the *x* direction), and full relaxation of all cell parameters.

GB Model	# of Atoms	$l_{z}(Å)$	Excess Volume/Area (Å)			GB Energy (J/m^2^)			Bond Range (Å)		
			No Relax	Only *x*	Full Relax	No Relax	Only *x*	Full Relax	No Relax	Only *x*	Full Relax
∑3(111)	48	13.7	0.00	0.06	0.01	0.05	0.05	0.05	0.01	0.01	0.02
$\sum 3\left(11\bar{2}\right)$	44	19.4	1.61	0.48	0.38	0.99	0.67	0.67	0.69	0.75	0.75
∑5(210)	80	17.7	0.00	0.82	0.60	1.11	0.93	0.91	1.04	0.48	0.48
∑5(310)	72	12.5	1.25	0.71	0.57	1.01	0.89	0.89	0.72	0.92	0.71
$\sum 7\left(12\bar{3}\right)$	78	14.8	1.06	0.81	0.63	0.98	0.96	0.94	0.80	0.78	0.82
$\sum 7\left(41\bar{5}\right)$	80	25.6	1.22	0.90	0.70	1.05	1.03	1.02	0.82	0.77	0.79
$\sum 9\left(1\bar{2}2\right)$	68	23.7	1.32	0.76	0.55	0.90	0.81	0.81	0.72	0.69	0.71
$\sum 9\left(41\bar{1}\right)$	64	16.8	1.86	0.55	0.46	1.20	0.76	0.76	0.71	0.59	0.59
∑11(113)	88	26.2	0.00	0.35	0.19	0.32	0.30	0.27	0.26	0.24	0.22
$\sum 11\left(23\bar{3}\right)$	80	18.6	1.69	0.72	0.28	1.07	0.82	0.53	0.64	0.86	0.42
∑13(320)	100	28.5	1.10	0.61	0.45	0.80	0.75	0.74	0.62	0.57	0.57
∑13(510)	100	40.3	1.55	0.74	0.60	1.04	1.00	0.96	0.80	0.79	0.79
